# Supplementary figures and images for: Real-time and immediate effects of backward walking exercise on pain intensity and lumbopelvic movement control in individuals with chronic non-specific low back pain with lumbar flexion syndrome
Source: PLoS One. 2025 Sep 3;20(9):e0330609. doi: 10.1371/journal.pone.0330609 (PMC12407490; doi:10.1371/journal.pone.0330609)

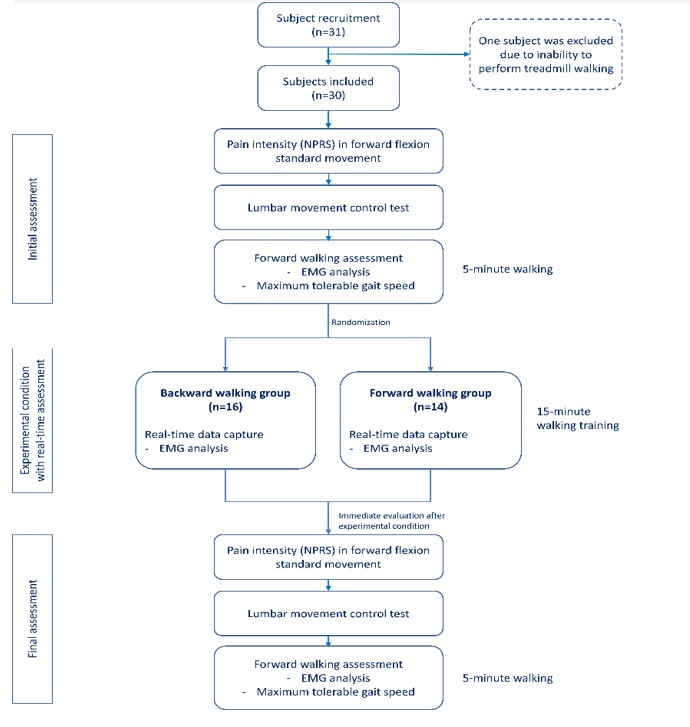

Supplement: S1 Fig — (TIF) [file pone.0330609.s001.tif]

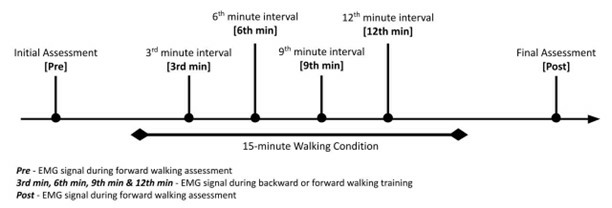

Supplement: S2 Fig — (TIF) [file pone.0330609.s002.tif]

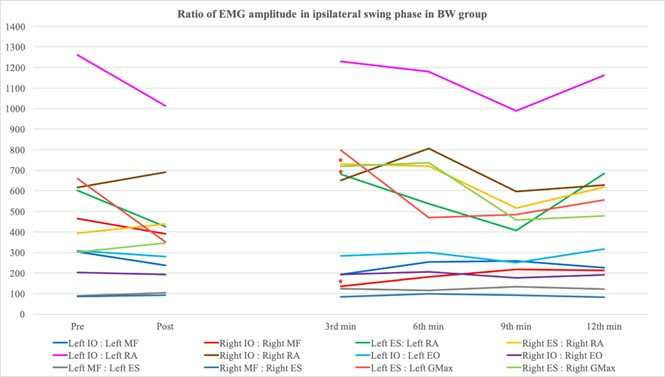

Supplement: S3 Fig — *: Significant changes compared with pre-training assessment (Bonferroni correction: p < 0.0083). (TIF) [file pone.0330609.s003.tif]

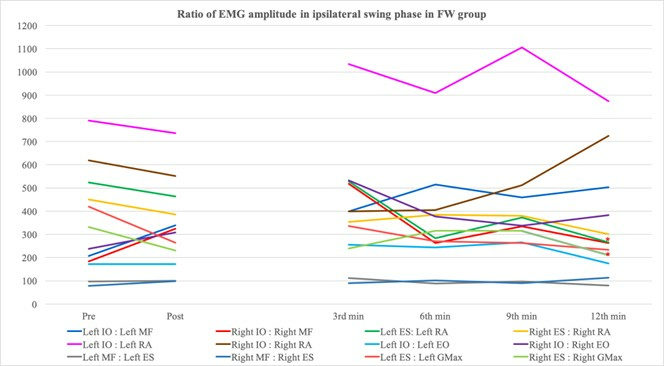

Supplement: S4 Fig — *: Significant changes compared with pre-training assessment (Bonferroni correction: p < 0.0083). (TIF) [file pone.0330609.s004.tif]

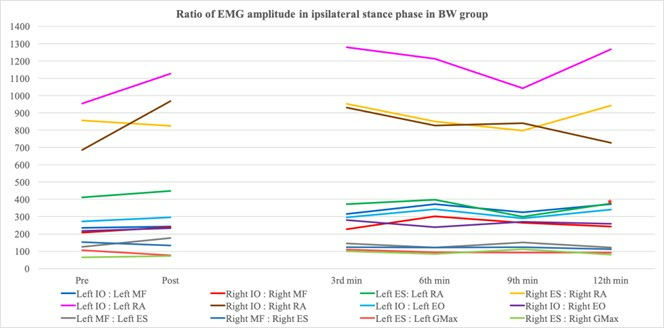

Supplement: S5 Fig — *: Significant changes compared with pre-training assessment (Bonferroni correction: p < 0.0083). (TIF) [file pone.0330609.s005.tif]

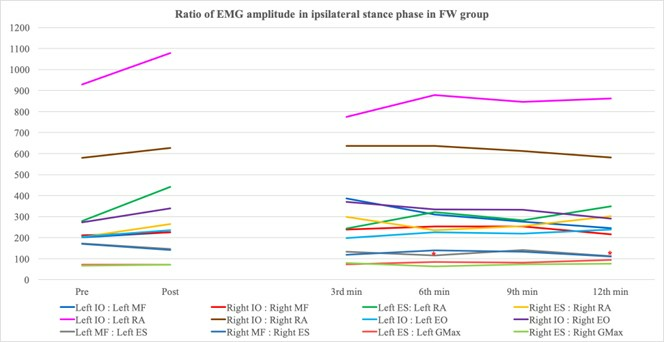

Supplement: S6 Fig — Significant changes compared with pre-training assessment (Bonferroni correction: p < 0.0083). (TIF) [file pone.0330609.s006.tif]
