# Supplementary material for: Real-time and immediate effects of backward walking exercise on pain intensity and lumbopelvic movement control in individuals with chronic non-specific low back pain with lumbar flexion syndrome
Source: PLoS One. 2025 Sep 3;20(9):e0330609. doi: 10.1371/journal.pone.0330609 (PMC12407490; doi:10.1371/journal.pone.0330609)
Supplement: Attachment — Submitted filename: Responses to reviewers_R2.docx [file pone.0330609.s010.docx]

**Manuscript title:** Real-time and immediate effects of backward walking exercise on pain intensity and lumbopelvic movement control in individuals with chronic non-specific low back pain with lumbar flexion syndrome

Manuscript ID: PONE-D-24-59535R2

**Reviewer #2:**

1. Thank you for the opportunity to re-evaluate the revised manuscript. I appreciate the authors’ thoughtful and detailed responses to my previous comments, as well as the substantive revisions made throughout the manuscript. The additions – including clarification of the sample size calculation, expanded discussion of generalizability, revised figures, and improvements to terminology and grammar – have significantly strengthened the paper. Most of my concerns have been addressed appropriately.

*Response: Thank you for your compliment and we are pleased to learn that our previous responses helped addressed most of the concerns raised in last round of review.*

2. However, I would like to request a few minor revisions prior to recommending acceptance: The inclusion of ICCs for all muscle groups is appreciated, and the discussion of the factors influencing the low reliability of the right erector spinae is thorough. However, I suggest that the main text (particularly in the Results and Discussion) include explicit cautionary language wherever findings involving this muscle are interpreted, to ensure that readers are aware of the limitations in reliability.

*Response: Thank you for your comments and recommendation. As advised, we have further revised our Results and Discussion by explicitly highlighting the precaution needed when interpreting the findings related to right erector spinae for its relatively low ICC as compared to the remaining list of muscles being investigated in this study. This now reads as, “The result indicated that all EMG measurements demonstrated good to excellent repeatability except right ES, and hence, interpretation of findings related to analysis of the right ES requires adequate caution for its observed variability of EMG magnitude.” (Lines 388-391, Results).*

*For Discussion section, it now reads as, “Although the level of reliability of right erector spinae EMG measurement is considered to be moderate with an ICC value of 0.558, it is indeed the lowest amongst the listed muscles. Therefore, interpretation of results involving EMG analysis of the right erector spinae would require extra caution irrespective to significant and insignificant findings statistically i.e., the significant increase in right ES:right GMax activity ratio during the 3^rd^ minute of BW training and significant decrease in the same ratio found during the 12^th^ minute of FW training, and those statistically insignificant comparisons reported in Tables 9, 12 and 13.” (Lines 626-632, Discussion)*

3. While the discussion now notes the potential for Type I error and small effect sizes, I still recommend that the authors slightly temper the language used in key interpretive statements.

*Response: Thank you for your comments and suggestion. Some refinement of the tone of our writing for the key interpretive statements was made with the aim to balancing the adequacy and accuracy of the discussion with respect to the potential Type I error and small effect size of our study (Lines 543, 547, 568, 603, 606, 650, 660-1, and 692).*

4. The revised data availability statement notes that data are available upon request due to the dataset’s complexity. For transparency, I suggest the authors clearly specify a contact point through which data can be requested, and ideally clarify what conditions apply (institutional approval, deidentified data only, etc.).

*Response: The data availability statement has been revised as, “Deidentified data are available upon request made to the corresponding author” (Entry at the online submission system).*

5. If these minor revisions are implemented, I would be pleased to support the manuscript’s acceptance.

*Response: We would like to thank the reviewer for the time and effort spent on reviewing our manuscript. We hope that we have adequately addressed the outstanding issues specified by the reviewer in this revised version. Thank you again for considering our revised manuscript.*

End of the response letter
